# Increase of chronic low back pain prevalence in a medium-sized city of southern Brazil

**DOI:** 10.1186/1471-2474-14-155

**Published:** 2013-05-01

**Authors:** Rodrigo D Meucci, Anaclaudia G Fassa, Vera MV Paniz, Marcelo C Silva, David H Wegman

**Affiliations:** 1 Post-Graduate Program in Epidemiology - Social Medicine Department, Federal University of Pelotas, Pelotas, Brazil; 2 Post-Graduate Program in Public Health, University of the Vale do Rio dos Sinos, Rio Grande do Sul, Brazil; 3 Superior School of Physical Education, Federal University of Pelotas, Pelotas, Brazil; 4 Department of Work Environment, University of Massachusetts Lowell, Lowell, USA

## Abstract

**Background:**

Chronic low back pain (CLBP) is a highly disabling morbidity with high social, economic and individual effects. Demographic, occupational and behavioral changes that took place in Brazil over the last decade are related with an increasing burden of chronic conditions. Despite these changes, comparison studies on CLBP prevalence and associated factors, over time are scarce in the literature in general, and unknown in Brazil. The present study compared the CLBP prevalence in a medium sized city in Brazil between the years 2002 and 2010 and examined factors associated with prevalence in 2010.

**Methods:**

Two cross-sectional studies with similar methodology were conducted in a medium-sized city in southern Brazil, in 2002 and 2010. 3182 individuals were interviewed in the first study and 2732 in the second one, all adults aged twenty years or more. Those who reported pain for seven weeks or more in the last three months in the lumbar region where considered cases of CLBP.

**Results:**

The CLBP prevalence increased from 4.2% to 9.6% in 8 years. In most of the studied subgroups the CLBP prevalence has at least doubled and the increase was even larger among younger individuals with more years of education and higher economic status.

**Conclusions:**

Increase in CLBP prevalence is worrisome because it is a condition responsible for substantial social impact, besides being an important source of demand for health services.

## Background

Low back pain is defined as a pain or discomfort located below the margin of the 12^th^ rib and above the inferior gluteal fold, with or without leg pain [1]. This is a very common complaint where, most of the time, resolution and return to work occurs in three months or less [2]. Most authors consider pain to be “chronic” when it endures three months or more [1,3-7]. However, some authors consider low back pain as chronic when it lasts seven weeks or more, while others require a duration of six months or more [8-12].

Regardless, chronic low back pain (CLBP) is a significantly disabling condition, responsible for long periods of absence from work. The greater this period the lower the chances of going back to work [13,14]. Moreover, CLBP is responsible for substantial workers’ compensation and social security expenses due to work absences and retirement [15-17].

Studies about the prevalence of CLBP use different case definitions, which makes it difficult to compare the findings. Studies using the criterion of pain lasting six months or more have shown a consistent prevalence around 15%,while those that defined CLBP as continuous pain for three months or more, have shown prevalence ranging from 4 to 24%. [1,4-7,11,12,18-21]. In addition to different CLBP definitions, variation in prevalence may be related to non-response rates, to methodological variability, to different cultural settings and to differences in prevalence over time [6,20].

In Brazil, two population-based studies have identified CLBP prevalence rates of 14.7% (in Salvador where the outcome was CLBP ≥ six months) and 4.2% (in Pelotas where the outcome was CLBP ≥ seven weeks (50 days) in the last three months). The prevalence difference in these two Brazilian communities is affected by different definitions, as well as ethnic and economic differences between the two cities. Salvador has a higher proportion of black people and people with low economic status than Pelotas which might also imply in different occupational profiles [10,11].

According to Freburger (2009), in the only comparison study on CLBP prevalence that used the same criterion and same methodology in two time periods, CLBP prevalence increased from 3.9% in 1992 to 10.2% in 2006 [1].

Demographic, occupational and behavioral changes that took place in Brazil over the last decade are associated with an increasing burden of chronic conditions. Despite these changes, comparison studies on CLBP prevalence and associated factors over time, including occupation, are scarce in the literature in general, and unknown in Brazil. In light of the importance of CLBP and the possibility of increasing prevalence, in 2010 a decision was made to repeat a population-based cross-sectional study of CLBP carried out in 2002. The objectives of the present study were: to examine change in CLBP prevalence over eight years in a middle income country using the same study methods; and to examine the trends according to demographic, occupational, socioeconomic and behavioral variables as well as factors related to CLBP.

## Methods

During the past decade, the Epidemiology Post-graduate Program at the Federal University of Pelotas has undertaken a series of population-based cross-sectional studies examining a variety of different health conditions, taking advantage of a postgraduate student research experience, known as the consortium. Briefly, the consortium is a shared fieldwork where all MSc students get data for their dissertations. Thus, this methodology incorporates a lot of research themes in the course of one multi-dimensional field study.

### Population

According to the 2000 Brazilian Census, the city of Pelotas was organized into 404 census tracks. Each census tracks had nearly 300 households. The two-stage sampling procedures were similar in both surveys. In order to obtain a sample of census tracks that represented a cross-section of socio-economic status, the census tracks were organized according to the average schooling of household head in 2002 and average income of household head in 2010. The census tracks were randomly selected proportionally according to size. Then, the households were randomly selected.

In 2002, 80 census tracks were selected and 20 households were systematically drawn from each sector. In 2010, 130 census tracks were selected and nearly 10 households were systematically drawn from each sector. Due to population growth in some areas of Pelotas, when the census tracks had more than 300 households, more than 10 households were selected. The change in the number of census areas in 2010 was a strategy to minimize the design effect of all outcomes being investigated in the consortium.

In both surveys, the residents of the sampled households aged 20 years or more with no physical or mental limitation to answering the questionnaire were considered eligible.

In 2002, in order to examine associated factors, the 3182 individuals interviewed were sufficient to obtain a statistical power of 80% and a confidence level of 95%, considering the same criteria used in 2010. To examine factors associated with CLBP in 2010 a sample size of 2842 was sought. The sample estimate took into account a confidence level of 95%, a statistical power of 80%, a exposed: nonexposed ratio of 1:1 female/male, a prevalence of CLBP in non-exposed of 3%, the ability to estimate prevalence risk ratios of 2,0 and the design effect of 1,4. It also included 10% for losses and refusals and 15% to account for multivariate modeling. This sample is larger than the necessary to compare the CLBP prevalence of 4.2% found in 2002 with the CLBP prevalence in 2010 and resulted in the ability to detect a ≥3% difference in prevalence with a confidence level of 95%.

### Surveys

**Figure 1:**
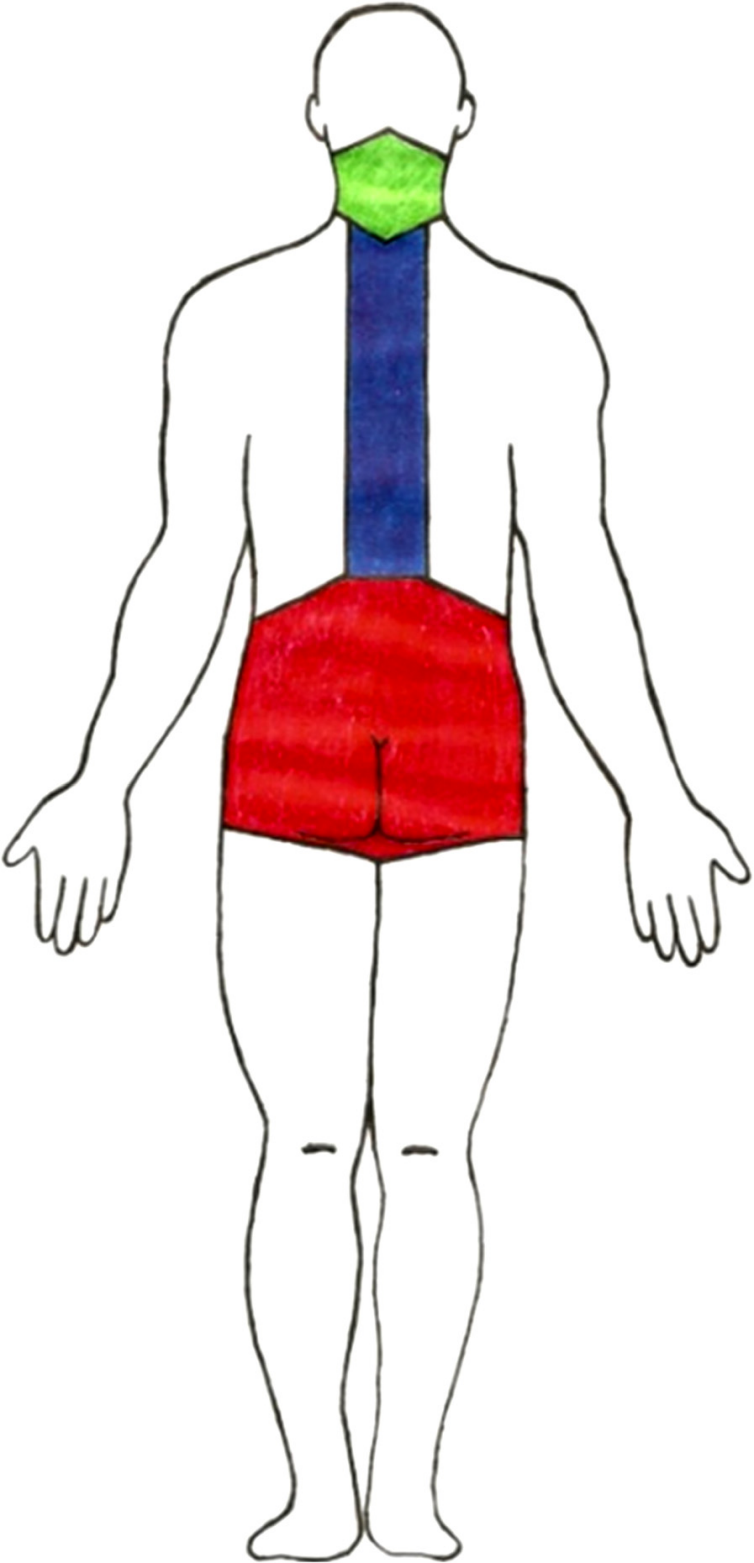
**Person in the supine and standing position with the lumbar, thoracic and cervical regions painted.** Legend: Green area: cervical region; Blue area: thoracic region; Red area: low back region.

A modified version of the original figures from the Standardized Nordic Questionnaire for musculoskeletal symptoms was used in both surveys [22]. A figure of a person in the supine and standing position with the lumbar, thoracic and cervical regions painted in different colors was presented to the interviewees (Figure 1).

The respondents who reported low back pain in the last year, identified as the lumbar region in the figure, answered the follow up question: "In the last three months, have you felt this pain for seven weeks or more (50 days) continuously? Subjects answering positively were considered to have CLBP.

In 2010, those with CLBP were additionally asked whether, in the past year, the pain limited daily living activities, resulted in paid work absenteeism or caused the individual to seek medical care from a physician. Demographic characteristics (sex, age, ethnicity, marital status), socioeconomic characteristics (education, economic status), behavioral characteristics (smoking, insufficient physical activity) and Body Mass Index (BMI) were collected in both surveys.

Age was collected in years and categorized in six age groups. Ethnicity was recorded as white, black, Asian, indigenous, and brown, but for the analyses it was dichotomized as white and non-white. Education was recorded as number of years of schooling and categorized in five subgroups.

Economic status was based on the Brazilian Criterion of Economic Classification that groups the population based on a combination of ownership of goods and head of the family educational level. The classification is considered an estimate of family purchasing power [23]. The Brazilian population is classified in seven groups: A1, A2, B1, B2, C, D, and E. The seven were collapsed into three categories: High (A/B), Medium C, and Low (D/E) for the analyses.

Cigarette smoking was recorded as non-smoker, ex-smoker or smoker. Respondents who reported smoking one cigarette or more a day were considered smokers; those who reported that stopped smoking for ≥30 days were considered as ex-smokers. Unlike the 2002 study, in which the BMI was calculated from self-reported weight and height, in 2010 the BMI was calculated from weight and height measured directly.

In 2002 the level of physical activity was assessed for leisure, work, commuting and at home. In 2010, only information on leisure time physical activity was collected. Both surveys used the short version of the International Physical Activity Questionnaire (IPAQ). Participants with scores below 150 minutes were classified as insufficiently active [24].

Respondents who reported working or having worked at some time in their life were asked about the main occupation they performed at the time of the interview or, if currently not employed, the main occupation in the past. For defining the main occupation, the following criteria were used: weekly hours of work or the occupation performed for the longest time or the one with the highest income. Main occupations were grouped as follows: never worked, trade/sales, education, health, industry, services in general, agriculture, cleaning services and civil construction.

Exposure to types of physical workloads were self-reported by participants in 2002 and 2010. In both years each respondent was asked about frequency of exposure to repetitive movements and to lifting or carrying weight. In 2010 participants reported on exposure specifically to forced/awkward postures and to static postures. No comparable questions were asked in 2002. Instead several survey items from 2002 were collapsed to represent a combination of forced/awkward or static postures (frequency of standing, sitting, kneeling, squatting, and lying). Frequency of all workload exposures in 2010 was classified as never, sometimes and always. Frequency in 2002 was classified for most exposures as never, rarely, usually, and always (for this study the latter two were collapsed into one category); exposure to repetitive movements was classified as yes/no.

In both studies fieldwork was conducted by interviewers who had completed at least high school and had received theoretical/practical training for this purpose. In 2010, the questionnaire was formatted and answers recorded on a Personal Digital Assistant. The anthropometrists used stadiometers with a maximum capacity of 2.0 meters and digital scales with a maximum capacity of 150 kilograms.

### Data analysis

Initially, a comparison by sex and age of the adult population of Pelotas was made between the 2000 and 2010 censuses and the 2002 and 2010 samples. The samples were then compared in terms of the other independent variables. Both survey analyzes were carried out taking into account the design effect of the studies.

The CLBP prevalence by survey (2002, 2010) was assessed according to the following variables: gender, age, skin color, education, socioeconomic status, smoking and BMI. Initially prevalence risk ratios and respective confidence intervals were calculated using Poisson regression without adjustment.

Multivariate hierarchical modeling was then carried out using the 2010 sample to examine the combined association of confounding or modifying factors on prevalence of CLBP [25]. We used Poisson regression with backward selection and the proposed model consisted of four levels. In the first, only gender, age and skin color were assessed; in the second, marital status and education were added; in the third, smoking, BMI (three categories) were added to the first two, and in the fourth insufficient physical activity and workloads were added along with all other factors. The variables with a p value ≤ 0.2 were kept in the model to control confounding factors. Variables with a p value < 0.05 were considered associated with CLBP. A separate model was developed using the variable ‘occupation’ along with all other variables except “education” since it was collinear with ‘occupation’.

The study was approved by the Ethics in Research Committee of the Federal University of *Pelotas*. All respondents signed an informed consent form, which contained the themes of the survey, the information confidentiality guarantee and the right to refuse the participation.

## Results

In the 2002 sample 1600 households and 3182 individuals were interviewed while in the 2010 sample 1352 households and 2732 individuals were interviewed. Refusals were 5.6% and 10.4% respectively [10]. The design effects found were 1.40 and 1.23 in 2002 and 2010 respectively.

In 2010, when BMI was measured directly, 19.0% did not participate in this part of the survey. The two samples appear representative of the general population with respect to age and sex (Table 1) [26]. The ratio male/female was 0.76 and 0.73 in 2002 and 2010 samples respectively. According to the census data, there was a reduction in the proportion of individuals aged 30–39 between 2000 and 2010 (22.7% to 19.3%). The age group 60–69 has increased its proportion from 10% to 11.4% (Table 1).

**Table 1:** Pelotas population according the 2000 and 2010 census and the surveys of 2002 and 2010

**Variable**		2000 Census^*^		**2002 Survey**		2010 Census^*^		**2010 Survey**
	**N**	**%**	**N**	%(CI 95%)^**^	**N**	**%**	**N**	%(CI 95%)^**^
**Sex**								
Male	97123	45,8	1374	43.2(41.5-44.9)	107757	45.6	1151	42.1(40.3-44.0)
Female	115016	54,2	1808	56.8(55.1-58.5)	128712	54.4	1581	57.9(56.0-59.7)
**Age**								
20-29	50620	23.9	719	22.6(21.1-24.1)	55415	23.4	595	21.8(20.2-23.3)
30-39	48196	22.7	680	21.4(19.9-22.8)	45696	19.3	462	16.9(15.5-18.3)
40-49	43759	20.6	667	21.0(19.5-22.4)	45363	19.2	545	20.0(18.4-21.4)
50-59	31283	14.7	533	16.8(15.4-18.0)	40231	17.0	495	18.1(16.7-19.6)
60-69	21190	10.0	307	9.6(8.6-10.7)	26959	11.4	369	13.5(12.2-14.8)
≥ 70	17091	8.1	276	8.6(7.7-9.7)	22805	9.7	266	9.7(8.6-10.8)

^*^Data from DATASUS (Database of the Unified Health System) according the Brazilian Census (Brazilian Institute of Geography and Statistics (IBGE).

^**^95% Confidence Interval.

Table 2 presents the frequencies of the remaining independent variables for both surveys. It shows that in the 2010 study there is a higher proportion of individuals with 12 or more years of study and in the Medium economic status (fewer in High status) than in 2002. Smoking decreased from 27.9% to 21.3% and obesity increased from 14.4% to 26% (Table 2).

**Table 2:** **Description of the studied samples in 2002 and 2010; *Pelotas, RS, *Brazil**

**Variable**		**2002**		**2010**
	**N**	%(CI 95%)**	**N**	%(CI 95%)**
**Skin color**				
White	2696	84.7(83.5-86.0)	2218	81.2(79.7-82.7)
Non white	486	15.3(14.0-16.5)	513	18.8(17.3-20.2)
**Education (years)**				
0	223	7.0(6.1-7.9)	184	6.7(5.8-7.7)
1-4	656	20.6(19.2-22.1)	494	18.1(16.6-19.5)
5-8	1067	33.6(31.9-35.2)	773	28.3(26.6-30.0)
9-11	780	24.5(23.0-26.0)	732	26.8(25.1-28.5)
≥ 12	451	14.2(13.0-15.4)	547	20.0(18.5-21.5)
**Economic status**				
High	747	23.6(22.1-25.0)	477	17.5(16.1-18.9)
Medium	1270	40.1(38.4-41.8)	1318	48.4(46.5-50.0)
Low	1153	36.3(34.7-38.0)	931	34.1(32.4-36.0)
**Civil status**				
Married/partner	1951	61.3(59.6-63.0)	1606	58.8(56.9-60.6)
Single/alone	1231	38.7(37.0-40.4)	1126	41.2(39.4-43.1)
**Occupation**				
Never worked	-	-	195	7.5(6.0-8.3)
Trade/sales	-	-	480	18.9(17.4-20.5)
Education	-	-	205	8.1(7.0-9.1)
Health	-	-	124	4.9(4.0-5.7)
Manufacturing	-	-	350	13.8(12.5-15.1)
Services	-	-	864	34.1(32.2-35.9)
Agriculture	-	-	66	2.6(2.0-3.2)
Cleaning	-	-	346	13.6(12.3-15.0)
Construction	-	-	99	3.9(3.1-4.7)
**Smoking**				
Never smoker	1668	52.4(50.7-54.2)	1495	54.7(52.8-56.6)
Former smoker	627	19.7(18.3-21.1)	654	24.0(22.3-25.5)
Current smoker	887	27.9(26.3-29.4)	583	21.3(19.8-22.9)
BMI*				
≤ 19.9	258	8.5(7.5-9.5)	115	4.7(3.9-5.5)
20-24.9	1284	42.1(40.4-43.9)	807	33.0(31.1-34.8)
25-29.9	1068	35.0(33.3-36.7)	888	36.3(34.4-38.2)
≥ 30	437	14.4(13.1-15.6)	638	26.0(24.3-27.8)
**Insufficient physical activity**^**§**^				
No	1837	58.9(55.1-62.7)	649	24.4(22.6-26.2)
Yes	1282	41.1(37.4-44.9)	2007	75.6(73.8-77.4)
**Weight lifting/loading**				
Never	-	-	975	38.5(36.6-40.4)
Sometimes	-	-	887	35.1(33.2-36.9)
Always	-	-	668	26.4(24.7-28.1)
**Repetitiveness**				
Never	-	-	387	15.3(13.9-16.8)
Sometimes	-	-	629	24.9(23.3-26.6
Always	-	-	1505	59.7(57.8-61.6)
**Forced/Awkward posture**				
Never	-	-	1072	42.4(40.4-44.3)
Sometimes	-	-	901	35.6(33.7-37.5)
Always	-	-	557	22.0(20.4-23.6)
**Static posture**				
Never	-	-	824	32.6(30.7-34.4)
Sometimes	-	-	842	33.3(31.4-35.1)
Always	-	-	863	34.1(32.3-36.0)
**Stopped doing some activity**^§§^				
No	-	-	154	48.4(42.9-54.0)
Yes	-	-	164	51.6(46.0-57.1)
**Sought medical attention**^§§^				
No	-	-	126	39.5(34.1-44.9)
Yes	-	-	193	60.5(55.1-65.9)
**Missed work**^||^				
No	-	-	114	80.3(73.7-86.9)
Yes	-	-	28	19.7(13.1-26.3)

^*^Body Mass Index.

^**^95% Confidence Interval.

^§^ In 2002 physical activity refers to leisure, work, commuting and at home; in 2010, physical activity was only assessed for leisure time.

^§§^ Data only for those who reported Chronic Low Back Pain in 2010.

^||^ Data only for those who reported Chronic Low Back Pain and were working in 2010.

The crude CLBP prevalence increased from 4.2% (95% CI 3.5-5.0) in 2002 to 9.6% (95% CI 8.3-0.8) in 2010, representing an increase of 129% (Table 3).

**Table 3:** **Chronic low back pain in adults of *Pelotas, *in the years 2002 and 2010**

**Variable**		** 2002**		** 2010**	
	**% (95% CI)**^**†**^	**p**	**% (95% CI)**^**†**^	**p**	**PRR (95% CI) **^||^
**Total**	4.2 (3.5-5.0)		9.6 (8.3-10.8)		2.29(1.77-2.68)
**Sex**		<0.001^**§**^		<0.001^**§**^	
Male	2.9 (2.3-3.5)		6.6 (5.1-8.1)		2.28(1.51-3.23)
Female	5.2 (4.4-6.0)		11.7 (10.0-13.4)		2.25(1.68-2.75)
**Age**		<0.001^**§§**^		<0.001^**§§**^	
20-29	1.0 (0.7-1.4)		3.9 (2.4-5.4)		3.90(1.56-7.79)
30-39	3.1 (2.5-3.8)		7.6 (5.2-10.0)		2.45(1.43-4.22)
40-49	5.3 (4.6-6.1)		10.8 (8.1-13.6)		2.04(1.29-2.94)
50-59	7.7 (6.8-8.7)		12.5 (9.3-15.7)		1.62(1.07-2.35)
60-69	4.9 (4.2-5.7)		13.0 (9.2-16.8)		2.65(1.48-4.71)
≥ 70	5.3 (4.6-6.1)		12.9 (8.6-17.2)		2.43(1.17-3.85)
**Skin color**		0.7^**§**^		0.25^**§**^	
White	4.3 (3.6-5.1)		9.3 (7.9-10.6)		2.16(1.66-2.62)
Non white	3.9 (3.2-4.6)		10.9 (8.1-13.7)		2.79(1.59-4.41)
**Education (years)**		<0.001^**§§**^		<0.001^**§§**^	
0	6.9 (6.0-7.8)		14.3 (9.7-18.9)		2.07(1.10-3.94)
1-4	6.3 (5.5-7.2)		13.0 (10.2-15.7)		2.06(1.33-2.89)
5-8	4.4 (3.7-5.2)		9.7 (7.5-11.9)		2.20(1.47-3.02)
9-11	2.7 (2.2-3.3)		8.1 (5.9-10.2)		3.00(1.75-4.67)
≥ 12	2.0 (1.5-2.6)		6.8 (4.7-8.8)		3.40(1.51-6.12)
**Economic status**		0.07^**§§**^		0.02^**§§**^	
High	2.8 (2.3-3.4)		7.8 (5.0-10.5)		2.79(1.48-4.20)
Medium	4.6 (3.9-5.4)		9.0 (7.4-10.5)		1.96(1.41-2.63)
Low	4.6 (3.9-5.4)		11.3 (9.0-13.6)		2.46(1.67-3.20)
**Smoking**		0.03^**§§**^		0.007^**§§**^	
Never smoker	3.2 (2.6-3.9)		8.0 (6.6-9.4)		2.50(1.68-3.14)
Former smoker	5.0 (4.3-5.8)		11.3 (8.5-14.1)		2.26(1.50-3.47)
Current smoker	5.5 (4.7-6.3)		11.5 (9.2-13.9)		2.09(1.41-2.94)
**BMI**^**‡**^		0.01^**§§**^		<0.001^**§§**^	
≤ 19.9	2.7 (2.1-3.3)		4.3 (0.5-8.0)		1.59(0.34-3.72)
20-24.9	3.4 (2.8-4.1)		8.0 (6.1-9.8)		2.35(1.60-3.43)
25-29.9	4.1 (3.4-4.9)		8.4 (6.5-10.2)		2.05(1.33-2.75)
≥ 30	6.2 (5.4-7.1)		14.2 (11.5-16.9)		2.29(1.44-3.37)

^**†**^ 95% Confidence Interval.

^**‡**^ BMI: Body Mass Index.

^§^ Chi-square test of heterogeneity.

^§§^Linear test for trend.

^||^ PRRs and CIs were estimated via poisson regression.

Among almost all categories of the seven analyzed variables, the CLBP prevalence has at least doubled, and except for one subgroup (BMI ≤19.9), all the results were statistically significant (Table 3).

The largest proportional increases were among the individuals aged 20–29 years, whose CLBP prevalence in 2010 was 3.9 times higher than in 2002, and among those with 12 years or more of education with CLBP prevalence 3.4 times higher in 2010 (Table 3).

In both surveys the prevalence of CLBP was greater in women, individuals with lower educational levels, smokers and obese individuals. There was no statistically significant difference in the prevalence of CLBP between whites and non-whites (Table 3).

In 2002, the prevalence of CLBP was greater among individuals in the 50–59 years age group (7.7%) and lower among the High economic status (2.8%), while Medium and Low economic status showed identical prevalence (4.6%) (Table 3).

In 2010, the individuals from the three older age groups (50–59, 60–69 and 70 years or older) showed similar prevalence of CLBP (12.5%, 13.0% and 12.9% respectively). Economic status was inversely associated with CLBP as low economic status presented a higher prevalence of CLBP when compared to Medium and High status (Table 3).

Table 4 presents the results of crude and adjusted analyses for the factors studied in the year 2010. By the crude analysis, gender, age, education, smoking, BMI, repetitiveness, weight lifting/loading and forced/awkward position were all associated with CLBP.

**Table 4:** Association of demographic, socioeconomic, behavioral, nutritional and workloads with chronic low back pain in 2010

**Variable**		**Crude analyses**			**Adjusted analyses**	
	**PRR**	**95% CI **^**†**^	**P**	**PRR**	**95% CI **^**†**^	**P**
**1**^**st **^**Level**						
**Sex**			<0,001^*^			<0,001^*^
Male	1	-		1	-	
Female	1.77	1.38-2.26		1.76	1.38-2.23	
**Age**			<0,001^**^			<0,001^**^
20-29	1	-		1	-	
30-39	1.96	1.19-3.22		1.96	1.20-3.21	
40-49	2.79	1.74-4.48		2.83	1.75-4.57	
50-59	3.23	1.99-5.25		3.24	1.99-5.25	
60-69	3.35	2.20-5.12		3.35	2.19-5.13	
70 or older	3.32	2.11-5.23		3.15	2.00-4.97	
**Skin Color**			0,3^*^			-
White	1	-		-	-	
Non White	1.18	0.89-1.56		-	-	
**2**^**nd **^**Level**						
**Civil Status**			0,04^*^			0,02^*^
Single/alone	1	-		1	-	
Married/partner	1.29	1.01-1.64		1.31	1.03-1.65	
**Education (y)**			<0,001^**^			0,01^**^
0	2.11	1.36-3.28		1.55	1.00-2.41	
1-4	1.92	1.37-2.68		1.46	1.03-2.07	
5-8	1.44	0.98-2.10		1.21	0.82-1.78	
9-11	1.19	0.78-1.81		1.20	0.79-1.83	
12 or more	1	-		1	-	
**3**^**rd **^**Level**						
**Smoking**			0,003^**^			0,005^**^
Never smoker	1	-		1	-	
Former smoker	1.41	1.05-1.90		1.23	0.90-1.69	
Current smoker	1.43	1.11-1.85		1.48	1.12-1.97	
**BMI**			<0,001^**^			0,01^**^
<25	1	-		1	-	
Overweight (25–29.9)	1.13	0.84-1.51		0.96	0.70-1.33	
Obesity (≥30)	1.91	1.43-2.53		1.50	1.12-2.01	
**4**^**th **^**Level**						
**Insufficient Physical Activity**			0.10^*^			0.90^*^
No	1	-		1	-	
Yes	1.30	0.95-1.79		1.02	0.74-1.43	
**Repetitiviness**			<0,001^**^			0,01^**^
Never	1	-		1	-	
Sometimes	1.51	0.97-2.35		1.47	0.89-2.41	
Always	2.05	1.38-3.05		1.70	1.09-2.64	
**Weight lifting/loading**			0,03^**^			0,73^**^
Never	1	-		1	-	
Sometimes	1.04	0.77-1.40		0.98	0.74-1.30	
Always	1.38	1.05-1.81		1.07	0.80-1.42	
**Forced/Akward position**			<0,001^**^			<0,001^**^
Never	1	-		1	-	
Sometimes	1.31	0.93-1.84		1.24	0.87-1.77	
Always	2.17	1.60-2.95		1.90	1.37-2.63	
**Static Posture**			0,15^**^			0,70^**^
Never	1	-		1	-	
Sometimes	1.29	0.96-1.73		1.04	0.77-1.40	
Always	1.27	0.92-1.77		0.94	0.66-1.36	

^*^ Wald Test for Heterogeneity.

^**^ Wald Test for Trend.

^**†**^ 95% Confidence Interval.

1st Level: adjusted between them; 2nd Level: adjusted between them and for the variables from the 1st level; 3rd Level: adjusted between them, for the ones from 1st and 2nd levels; 4th Level: adjusted between them and for previous levels.

‘Being female’ (PRR 1.76), ‘smoker’ (PRR 1.48) and ‘married’ (PRR 1.31) represented risk factors for CLBP. Increasing ‘Age’ and ‘BMI’ were linear positive associated with CLBP, while increasing ‘education’ was inversely associated. With regard to ‘workload’, there was a positive linear association with frequency of ‘repetitive movements’ and ‘forced/awkward position’ variables (Table 4). The ‘weight lifting/loading’ variable was not statistically significantly associated with CLBP.

Regarding occupation, the highest prevalence of CLBP was among agriculture workers (16.7%), followed by cleaning service workers (14.4%), and the lowest one was among individuals who have never worked (4.6%). According to the adjusted analysis, agriculture (PRR 2.78), services in general (PRR 2.30) and cleaning services (PRR 2.11) workers were at risk for CLBP (Table 5).

**Table 5:** Prevalence and association of occupation with chronic low back pain

**Variable**			** Crude analyses**				Adjusted analyses^**^	
	**N**	**%**	**PRR**	**95% CI **^**†**^	**P**	**PRR**	**95% CI**^**†**^	**P**
**Occupation**					0,001^*^			0,03^*^
Never Worked	195	4.6	1	-		1	-	
Trade/sales	480	6.3	1.35	0.65-2.82		1.36	0.62-3.00	
Health	124	8.9	1.91	0.82-4.47		1.53	0.62-3.75	
Education	205	8.3	1.79	0.77-4.14		1.66	0.71-3.89	
Manufacturing	350	10.0	2.16	1.00-4.64		2.07	0.94-4.54	
Cleaning	346	14.4	3.11	1.49-6.49		2.11	1.00-4.54	
Services	864	10.3	2.23	1.15-4.33		2.30	1.13-4.65	
Civil construction	99	9.1	1.96	0.79-4.88		2.47	0.92-6.64	
Agriculture	66	16.7	3.59	1.43-9.01		2.78	1.03-7.51	

^*^ Wald Test for Heterogeneity.

^**^Adjusted for the variables from 1st, 2nd and 3rd levels.

^**†**^ 95% Confidence Interval.

Activity limitation due to CLBP was common with half of the individuals with CLBP stopping some kind of activity. In addition 60% sought medical attention in the last year and 20% of those working missed work due to CLBP (Table 2).

## Discussion

Our study results compared with findings from 8 years previously show an increase in the prevalence of CLBP that occurred in almost every category studied. Surprisingly, the greatest proportional increases occurred among younger and more educated individuals. ‘Being a female’, ‘smoker’, and ‘married’ were considered as risk factors for CLBP. ‘Age’, ‘BMI’, ‘exposure to repetitive movements and to awkward position at work’ had a positive linear association with CLBP, while ‘weight lifting/loading’, associated with CLBP in 2002, was not found important in 2010. The educational level showed an inverse association with CLBP, and workers in agriculture, general services and cleaning services presented higher risk of CLBP when compared with non-workers.

The increased prevalence of CLBP may be explained partly by the aging of the population of Pelotas – the 2002 sample included 44% <40 years old while the 2010 sample had only 38.7% <40 years old.

Although the greatest proportional increase in the prevalence of CLBP was among younger individuals, increasing age presented a positive linear association with the outcome in both surveys. The age-related degenerative processes that occur in the articular structures of the lumbosacral spine are considered as factors that contribute to the development of CLBP [4,8,10,18].

The inverse relationship between education and CLPB in 2010 is consistent with the 2002 survey [10]. Furthermore, literature suggests that less educated individuals are exposed to occupations with higher risk for injuries in low back [10,18].

In both surveys women were at higher risk of reporting CLBP than men [1,10]. Anatomical and physiological characteristics predispose women to CLBP when compared to men. Furthermore, women are increasingly included in the labour market, adding occupational workloads to the ones associated with housework, including childcare, and paidwork [10].

Being married/living with a partner was found as a risk factor for CLBP in both surveys. The reasons for this finding are uncertain, and this variable is probably a risk marker for occupational and home exposures [10].

According to our findings, smokers are at a higher risk of reporting CLBP. Nicotine may reduce bloody perfusion to intervertebral discs and increase proinflammatory cytokines levels that potentiates pain transmission in the central nervous system [10,18,27].

The association between obesity and CLBP observed in both surveys along with the increased prevalence of obesity may be another factor contributing to the increase in CLBP. The proportion of the sample with BMI ≥25 increased from 49% to 62%. Although the method for assessing BMI changed between surveys, population studies suggest that the magnitude of the difference in self-report and measured height and weight is small although the self-report generally underestimates BMI [28,29]. Furthermore, another population-based study conducted in Pelotas in 2000 used anthropometric measures to assess BMI. It found prevalence of BMI ≥30 to be 19.4% [30], which was somewhat larger than the self-reported prevalence found in 2002. Obesity is associated with chronic low back pain probably related to the overload of the articular structures of the lumbosacral spine [1,31].

Changes in the labor market of Pelotas may also contribute to the increase in CLBP in 2010. Although the 2002 survey did not collect information on main occupation, official statistics provide some supporting evidence. The service and trade sectors were responsible for the majority of formal employment in both periods [31]. Moreover, proportional employment in the services sector increased from 33.1% to 38.8% and in the trade sector from 22.4% to 26.0%. Civil construction showed a slight increase, while the sectors of manufacturing, public administration and activities related to the primary sector of the economy experienced reductions from 18.8% to 13.0%; 17.1% to 13.8% and 2.8% to 1.8%, respectively [32].

The service sector is mainly characterized by exposure to repetitive movements and permanence in awkward positions. Repetitive movements were associated with CLBP in both surveys, while permanence in awkward position (not collected in 2002) was associated in 2010. This sector is characterized by the demand for better skilled labor, which can be related to the important increase in CLPB among younger and more educated individuals.

The reduction in number of jobs in some sectors is related to economic restructuring that began in the 1990’s. This process occurred in all economic activities and resulted in the intensification of work, loss of autonomy, reduction of break time, and increase in awkward postures and repetitive movements [33]. Such changes may have affected the respondents’ perception regarding the studied workloads. This may have occurred in relation to the ‘weight lifting/loading’ variable, which was only associated with CLBP in 2002. The automation process and the predominance of activities related to services and trade reduced the practice of ‘weight lifting’ at work. Thus, it is possible that in 2010 the respondents have considered to be exposed to ‘weight lifting/loading’ while loading less weight than in 2002.

In addition to occupational exposures, other behavioral changes, such as the intensive use of computers and always being in awkward postures may have contributed to the increased prevalence of CLBP.

The fact that more than a half of the individuals in 2010 with CLBP had stopped certain physical activity due to pain, along with the absenteeism reported among individuals who had paying jobs, suggests that this morbidity has significant impacts upon the daily activities of those people. However, the demand for medical attention was lower than seen in the North Carolina study [1]. The reasons for this lower demand may be related to the fact that care seeking among patients with CLBP is linked to social security issues, such as sick leave. Furthermore, in Brazil there is a large contingent of informal workers who may need to manage their chronic condition without seeking for medical care.

Another study from Pelotas found a chronic back pain prevalence of 18.9% (neck, upper back and low back) [34]. Despite methodological differences, these findings suggest that chronic musculoskeletal pain is a public health problem which burden has to be understood.

This study has limitations that should be noted. Because the data are based on two cross-sectional studies, the possibility of reverse causality in the analysis of associated factors should be considered, especially in relation to physical inactivity and workloads. The associations might be affected by the healthy worker effect. The study recorded the main occupation each subject has or had (for those not currently working), and workers with CLBP might migrate from occupations related with CLBP to less demanding occupations regarding musculoskeletal system. Another important limitation is the absence of information, in either 2002 or 2010 on whether work was in the formal or informal sector. Other concern is the fact that the study did not specified repetitive movements, forced/awkward postures and static postures regarding low back movements, thus this workloads could be connected to upper limbs movements. Nevertheless, for further studies, it is important to evaluate these workloads in depth.

As strengths, this is the first Brazilian study that compared the prevalence of CLBP in the same community using similar methodology and that had described and analyzed occupation and its association to CLBP.

## Conclusions

This study adds to the understanding of CLBP progression. Our findings highlights that CLBP prevalence should be monitored and poses a challenge in research agenda in developing countries, since CLBP is known as a condition responsible for substantial social impact, besides being an important source of demand for health services.

## Abbreviations

CLBP: Chronic low back pain; BMI: Body mass index; IPAQ: International physical activity questionnaire; PRR: Prevalence risk ratio.

## Competing interests

The authors declare that they have no conflicts of interest.

## Authors’ contributions

RDM wrote the 2010 project focused on comparison with 2002, performed the bibliographic review, coordinated the fieldwork, developed data analyses, and wrote the article. AGF oversaw the 2002 and 2010 projects preparation, data analyses and the manuscript preparation. VMVP participated in designing the 2010 project, data analyses and manuscript preparation. MCS wrote and coordinated the field work of the 2002 project and contributed to the data analysis and manuscript preparation. DHW provided advice on data analysis and manuscript preparation. All authors read and approved the final manuscript.

## Pre-publication history

The pre-publication history for this paper can be accessed here:

http://www.biomedcentral.com/1471-2474/14/155/prepub
